# Author Correction: Genetic alterations in the 3q26.31-32 locus confer an aggressive prostate cancer phenotype

**DOI:** 10.1038/s42003-020-01291-8

**Published:** 2020-10-08

**Authors:** Benjamin S. Simpson, Niedzica Camacho, Hayley J. Luxton, Hayley Pye, Ron Finn, Susan Heavey, Jason Pitt, Caroline M. Moore, Hayley C. Whitaker

**Affiliations:** 1 Molecular Diagnostics and Therapeutics Group, Research Department of Targeted Intervention, Division of Surgery & Interventional Science, University College London, London, UK; 2 Human Oncology and Pathogenesis Program, Memorial Sloan Kettering Cancer Center, New York, NY USA; 3 Marie-Josée and Henry R. Kravis Center for Molecular Oncology, Memorial Sloan Kettering Cancer Center, New York, NY USA; 4 Cancer Institute of Singapore, National University of Singapore, Singapore, Singapore; 5 Department of Urology, UCLH NHS Foundation Trust, London, UK

**Keywords:** Genomic instability, Cancer genomics

Correction to: *Communications Biology* 10.1038/s42003-020-01175-x, published online 14 August 2020.

In the original version of the published Article, the following affiliation was incorrectly included for author Niedzica Camacho: “Department of Pathology, Memorial Sloan Kettering Cancer Center, New York, New York for Genomics Research, Discovery Sciences, Biopharmaceutical R&D, AstraZeneca, Cambridge, UK”. This affiliation has been removed in the PDF and HTML versions of the Article.

