# Supplementary material for: Genetic alterations in the 3q26.31-32 locus confer an aggressive prostate cancer phenotype
Source: Commun Biol. 2020 Aug 14;3:440. doi: 10.1038/s42003-020-01175-x (PMC7429505; doi:10.1038/s42003-020-01175-x)
Supplement: Supplementary file 1 — Supplementary Information [file 42003_2020_1175_MOESM1_ESM.pdf]

Supplementary information for "Genetic alterations in the 3q26.31-32 locus confer an aggressive prostate cancer phenotype"

Simpson et al

a

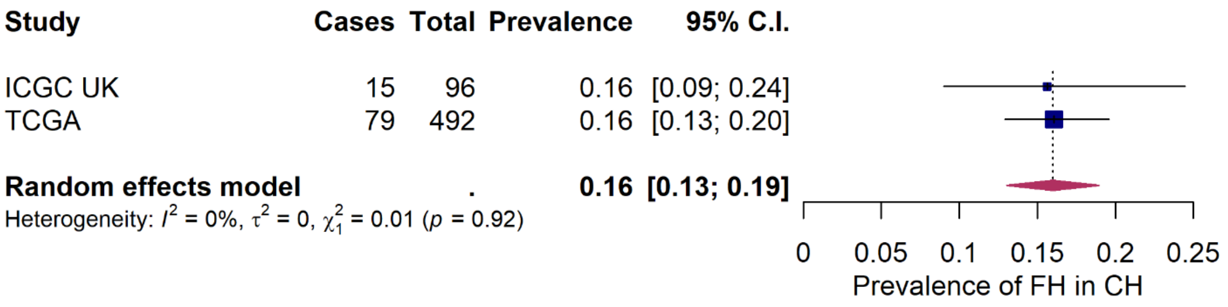

b

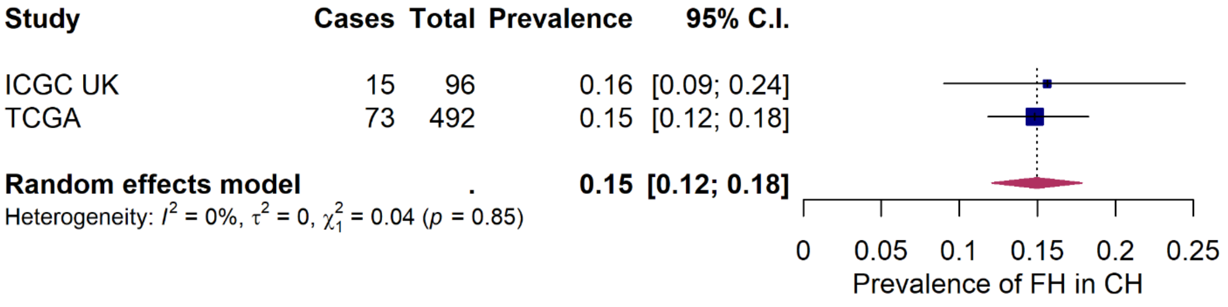

Supplementary Figure 1. Summary of the estimated frequency of NAALADL2 and TBL1XR1 gains and amplifications. Figure shows of the number of gains/amplifications (Gain/Amp), the total number of patients in the ICGC UK, ICGC Canada (CA) and TCGA cohorts with corresponding forrest plots displaying the proportion (and upper and lower 95% confidence intervals) of patients with this alteration for: A) NAALADL2 and B) TBL1XR1. Each plot also displays a summary proportion (pink diamond) estimated by a random-effects model. Also displayed are measures of study heterogeneity such as I^2 τ^2 and χ^2_2.

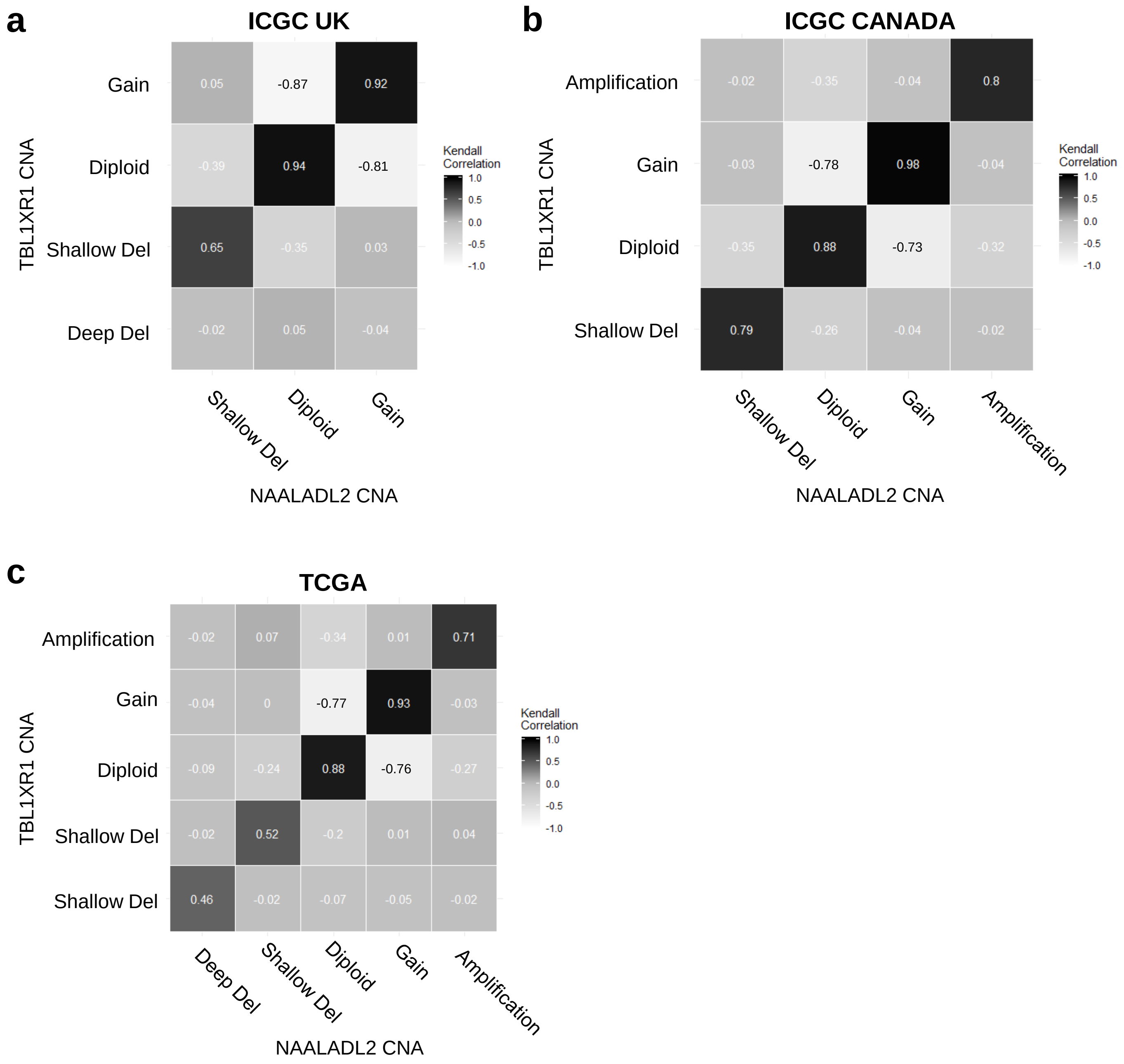

Supplementary Figure 2. Co-occurrence heatmaps for copy-number alterations of NAALADL2 compared to TBL1XR1. Plotted is a heatmap displaying where colour intensity corresponds to the Kendalls correlation coefficient, between 1(black) and -1 (white), for the bivariate comparison of each class of copy-number alteration in: A) the ICGC UK cohort, B) the ICGC Canada and C) the TCGA cohort. Only alteration types present in each cohort are displayed in the comparison.

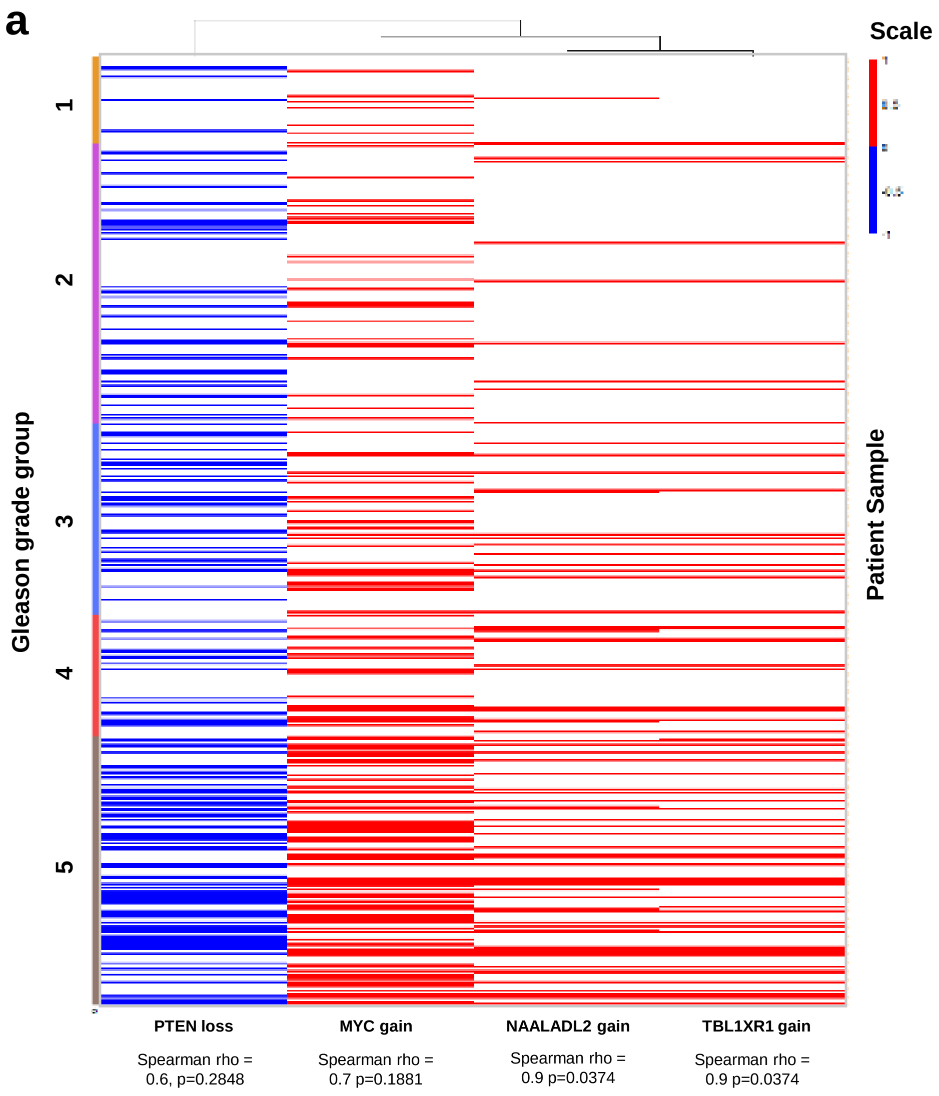

Supplementary figure 3. Frequency of NAALADL2/TBL1XR1 gains/amplifications across Grade Groups. A) Heatmap displaying frequency of PTEN loss, MYC gain, NAALADL2 gain and TBL1XR1 gain across Grade groups in the TCGA cohort (n=498). P values show the result of Spearman's rho correlation between Grade Group and alteration frequency.

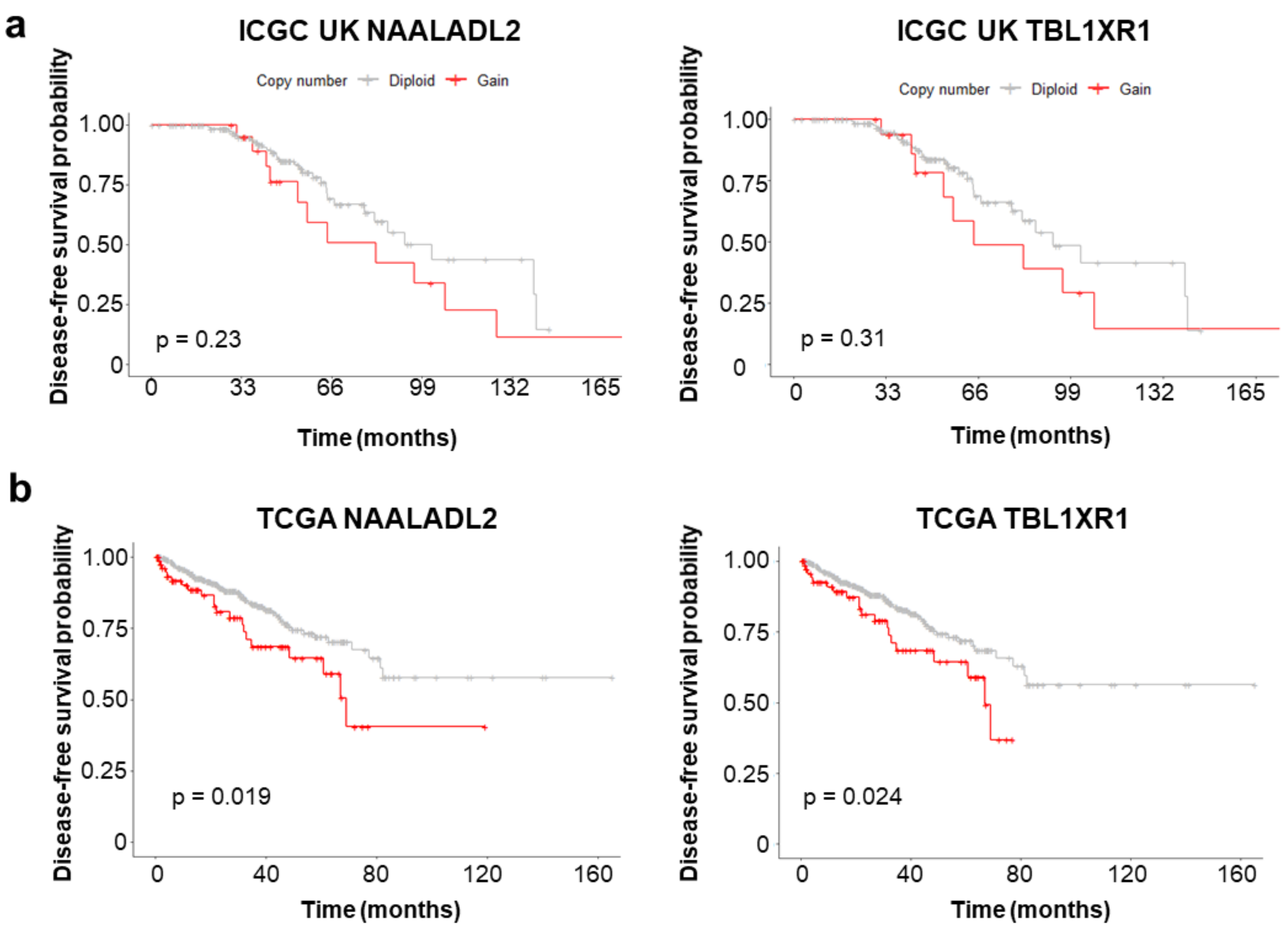

Supplementary figure 4. *Disease-free survival of patients with NAALADL2/TBL1XR1 gains/amplifications. Survival curves are represented using the Kaplan-Meier method, and survival rates were compared with the use of the two-sided log-rank (Mantel-Cox) test. A) Disease-free survival of patients following radical prostatectomy in the ICGC UK cohort (n=99). B) Disease-free survival in the TCGA cohort following radical prostatectomy (n=498).*

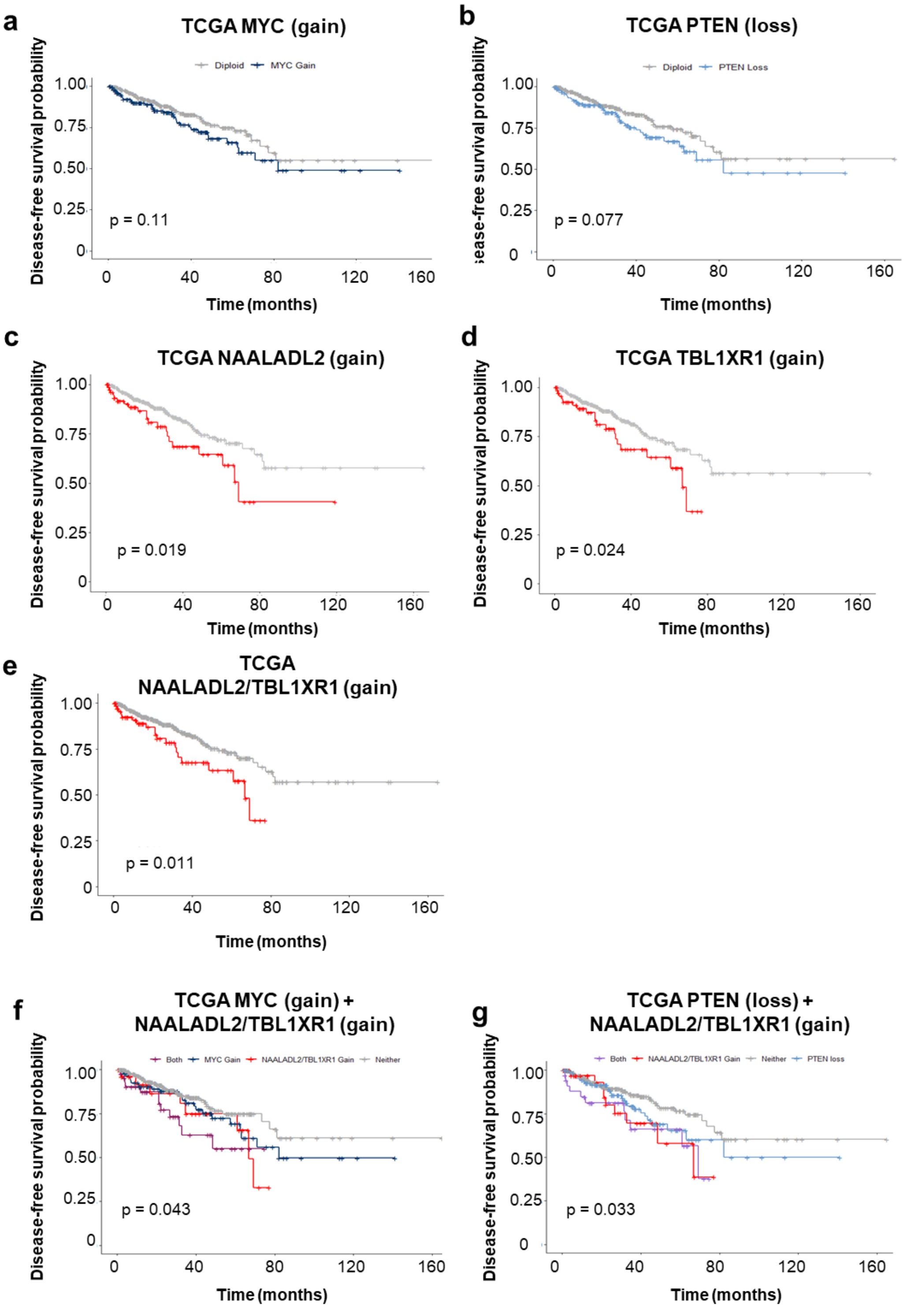

Supplementary Figure 5. Association with disease-free survival of patients with copy-number loss of PTEN and MYC gain compared to NAALADL2 and TBL1XR1 in the TCGA. Shown is disease-free survival in primary prostate cancer following radical prostatectomy in the TCGA cohort, with patients segregated based on: A) PTEN loss (hetero or homozygous loss), B) MYC gain/amplification, C) NAALADL2 gain/amplification, D) TBL1XR1 gain/amplification, E) NAALADL2 and TBL1XR1 loss compared to diploid (grey). F) Patients with only; PTEN loss, NAALADL2/TBL1XR1 gain or both compared to diploid (mutually exclusive groups), G) MYC gain, NAALADL2/TBL1XR1 gain or both compared to diploid (grey) whereby all groups are mutually exclusive of one another. P-value shows the result of a Log-Rank (Mantel-Cox) test.

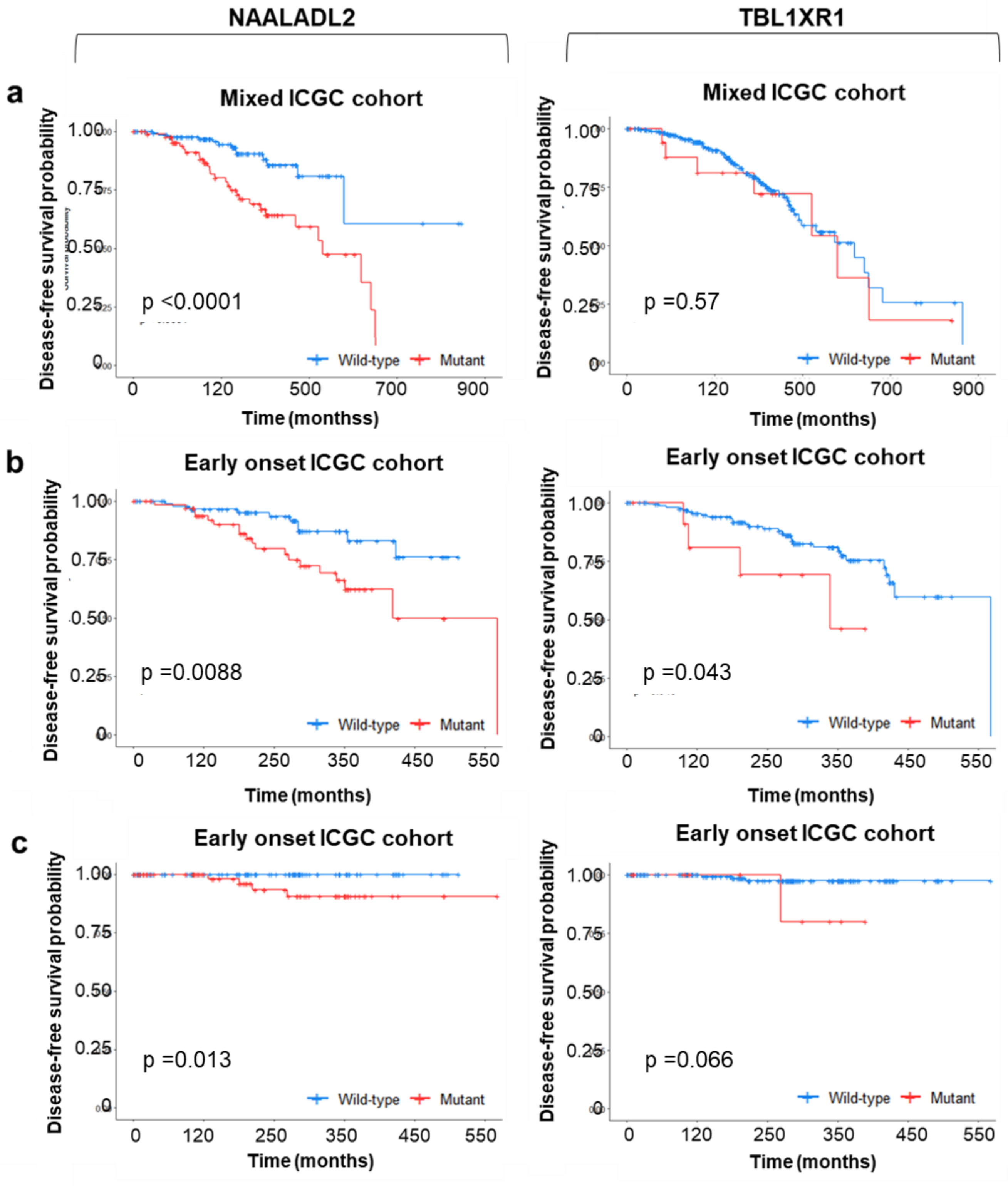

Supplementary Figure 6. Single base alterations in NAALADL2 and TBL1XR1 associate with prognosis in two ICGC cohorts. A) Disease-free survival in primary prostate cancer following radical prostatectomy in a pooled ICGC cohort (UK, Canada, China) for patients with single base alterations in NAALADL2 and TBL1XR1. B) Disease-free survival in early-onset primary prostate cancer patients following radical prostatectomy in the ICGC Denmark cohort (EOPC) for NAALADL2 and TBL1XR1. C) Overall survival in early-onset primary prostate cancer patients following radical prostatectomy in the ICGC Denmark cohort (EOPC) for NAALADL2 and TBL1XR1. P values show the result of a log-rank (Mantel-Cox) test.

a

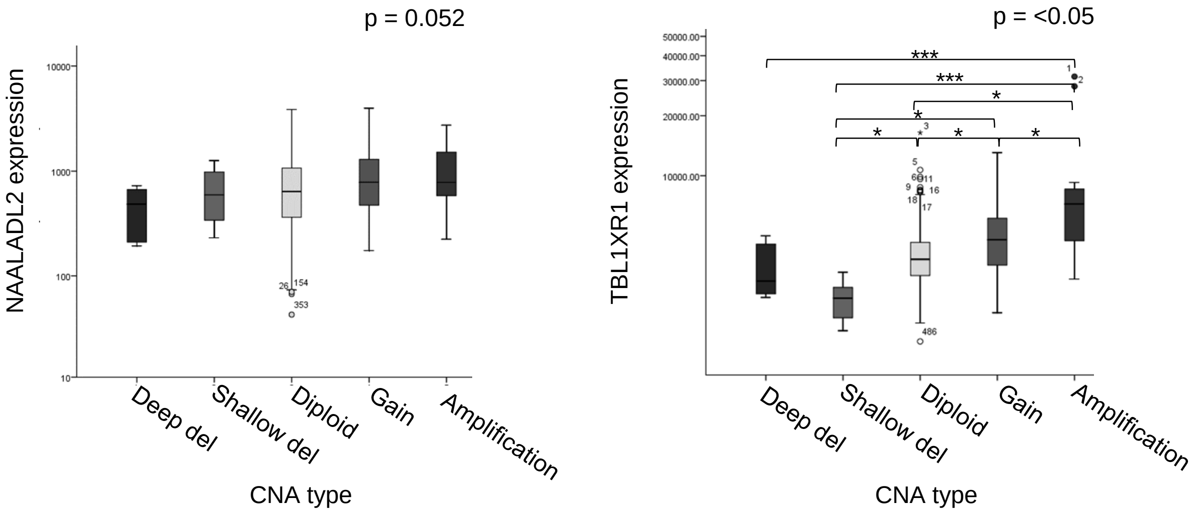

Supplementary Figure 7. NAALADL2 and TBL1XR1 expression is associated with copy-number status in the TCGA. Boxplots show mRNA expression for A) NAALADL2 and B) TBL1XR1 across patients with copy-number gain/amplification in the same gene. P values show the results of an ANOVA or ANOVA followed by Tukeys test. Center lines indicate median values, box limits represent first and third quartiles amd whiskers display the minimum and maximum expression values (RSEM).
