# Supplementary material for: Genetic alterations in the 3q26.31-32 locus confer an aggressive prostate cancer phenotype
Source: Commun Biol. 2020 Aug 14;3:440. doi: 10.1038/s42003-020-01175-x (PMC7429505; doi:10.1038/s42003-020-01175-x)
Supplement: Supplementary file 2 — Description of Additional Supplementary Files [file 42003_2020_1175_MOESM2_ESM.pdf]

Description of Additional Supplementary Files

Supplementary Data 1: alteration_frequency_in_PCa_cohorts

Somatic alterations in NAALADL2 and TBL1XR1 and their frequencies across prostate cancer cohorts from cbioportal (full list of included cohorts in appendix 1.

Supplementary Data 2: Gain_Co-occurance_in_3_cohorts

The genome wide co-occurrence (calculated using a Fisher's exact test) of copy-number gains with gains in NAALADL2 in three cohorts. All significant gains defined using GISTIC 2.

Supplementary Data 3: Co-amplified_oncogenes

Significantly co-amplified genes in all three cohorts which are identified as oncogenes by The Network of Cancer Genes (NCG) database.

Supplementary Data 4: Clinical_data

Clinical data from the TCGA used to compare clinical associations with gains in NAALADL2 and TBL1XR1.

Supplementary Data 5: Multivariable_Cox_regressions_TCGA

Outputs from multivariable cox regression models to predict disease-free survival in the TCGA cohort.

Supplementary Data 6: DEG_analysis_DESeq2

The output of DESeq2 comparing differentially expressed genes between patients with and without gains in NAALADL2 and TBL1XR1.

Supplementary Data 7: Oncogenes_present_in_DEG_list_overlap

Differentially expressed genes which overlap between NAALADL2 and TBL1XR1 which are identified as oncogenes by The Network of Cancer Genes (NCG) database.

Supplementary Data 8: Heatmap_z_scores

Scaled and centred (z scores) expression of genes altered in patients with gains in NAALADL2 and TBL1XR1 used to create the heatmap shown in figure 3.

Supplementary Data 9: GSEA_for_NAALADL2_TBL1XR1_DEGs

Gene set enrichment analysis for DEGs between those with and without gains in NAALADL2 or TBL1XR1.

Supplementary Data 10: ORA_Analysis_for_shared_DEGs

Overrepresentation analysis performed on the overlapping DEGs for NAALADL2 gains and TBL1XR1 gains.
