# Supplementary material for: Genetic alterations in the 3q26.31-32 locus confer an aggressive prostate cancer phenotype
Source: Commun Biol. 2020 Aug 14;3:440. doi: 10.1038/s42003-020-01175-x (PMC7429505; doi:10.1038/s42003-020-01175-x)
Supplement: Supplementary file 13 — Reporting Summary [file 42003_2020_1175_MOESM13_ESM.pdf]

nature research

Corresponding author(s): Hayley C. Whitaker

Last updated by author(s): 2020/05/30

# Reporting Summary

Nature Research wishes to improve the reproducibility of the work that we publish. This form provides structure for consistency and transparency in reporting. For further information on Nature Research policies, see Authors & Referees and the Editorial Policy Checklist.

## Statistics

For all statistical analyses, confirm that the following items are present in the figure legend, table legend, main text, or Methods section.

| n/a | Confirmed | |
|---|---|---|
| ☐ | ☒ | The exact sample size (*n*) for each experimental group/condition, given as a discrete number and unit of measurement |
| ☐ | ☒ | A statement on whether measurements were taken from distinct samples or whether the same sample was measured repeatedly |
| ☐ | ☒ | The statistical test(s) used AND whether they are one- or two-sided<br>*Only common tests should be described solely by name; describe more complex techniques in the Methods section.* |
| ☐ | ☒ | A description of all covariates tested |
| ☐ | ☒ | A description of any assumptions or corrections, such as tests of normality and adjustment for multiple comparisons |
| ☐ | ☒ | A full description of the statistical parameters including central tendency (e.g. means) or other basic estimates (e.g. regression coefficient) AND variation (e.g. standard deviation) or associated estimates of uncertainty (e.g. confidence intervals) |
| ☐ | ☒ | For null hypothesis testing, the test statistic (e.g. *F*, *t*, *r*) with confidence intervals, effect sizes, degrees of freedom and *P* value noted<br>*Give P values as exact values whenever suitable.* |
| ☒ | ☐ | For Bayesian analysis, information on the choice of priors and Markov chain Monte Carlo settings |
| ☒ | ☐ | For hierarchical and complex designs, identification of the appropriate level for tests and full reporting of outcomes |
| ☒ | ☐ | Estimates of effect sizes (e.g. Cohen's *d*, Pearson's *r*), indicating how they were calculated |

*Our web collection on statistics for biologists contains articles on many of the points above.*

## Software and code

Policy information about availability of computer code

| | |
|---|---|
| Data collection | No software was used |
| Data analysis | All code used for data analysis in this manuscript was performed using commercial or open source code already published in the literature: software included:R version 3.3.1 (packages: Dplyr, ggplot, survminer, GOPlot, DESeq2, heatmapper), IBM SPSS statistics 24 v22.0 and GISTIC2 (performed using the genepattern platform created by the broad institute). |

For manuscripts utilizing custom algorithms or software that are central to the research but not yet described in published literature, software must be made available to editors/reviewers. We strongly encourage code deposition in a community repository (e.g. GitHub). See the Nature Research guidelines for submitting code & software for further information.

## Data

Policy information about availability of data

All manuscripts must include a data availability statement. This statement should provide the following information, where applicable:

- Accession codes, unique identifiers, or web links for publicly available datasets
- A list of figures that have associated raw data
- A description of any restrictions on data availability

The datasets generated during and/or analysed during the current study are available in the following repositories: Somatic alterations in meta-cohort from cbioportal: https://www.cbioportal.org/, ICGC cohorts: https://dcc.icgc.org/, TCGA PRAD:http://firebrowse.org/?cohort=PRAD&download_dialog=true. Full links are also provided in the materials and methods section. Please contact the corresponding author for any other queries.

# Field-specific reporting

Please select the one below that is the best fit for your research. If you are not sure, read the appropriate sections before making your selection.

☒ Life sciences ☐ Behavioural & social sciences ☐ Ecological, evolutionary & environmental sciences

For a reference copy of the document with all sections, see nature.com/documents/nr-reporting-summary-flat.pdf

# Life sciences study design

All studies must disclose on these points even when the disclosure is negative.

| | |
|---|---|
| Sample size | In this study we utilized The Cancer Genome Atlas and International Cancer Genome Consortium cohorts, therefore no sample size calculation was performed as these are already published and, at the time of publication, were the largest prostate cancer studies available which included somatic copy-number and whole transcriptome data with suitable clinical follow-up. There are no more suitable cohorts in existence that the authors know of to perform this analysis. |
| Data exclusions | No data were excluded throughout this analysis unless the variable of interest (i.e. copy-number or mRNA expression) was absent. All reported n values describe the number of included samples used in the analysis in opposed to the full number of patients in each cohort. |
| Replication | All data is publicly available and can be repeated by any researcher whom wishes to do so. All data was subject to routine 'sanity checks' to ensure its veracity. |
| Randomization | As this study was primarily observational in nature and looked at genetic and clinical phenotypes there was no need for the use of randomization in this study. |
| Blinding | As this study was primarily observational in nature and looked at genetic and clinical phenotypes there was no need for the use of blinding in this study. |

# Reporting for specific materials, systems and methods

We require information from authors about some types of materials, experimental systems and methods used in many studies. Here, indicate whether each material, system or method listed is relevant to your study. If you are not sure if a list item applies to your research, read the appropriate section before selecting a response.

## Materials & experimental systems

| n/a | Involved in the study | |
|---|---|---|
| ☒ | ☐ | Antibodies |
| ☒ | ☐ | Eukaryotic cell lines |
| ☒ | ☐ | Palaeontology |
| ☒ | ☐ | Animals and other organisms |
| ☒ | ☐ | Human research participants |
| ☒ | ☐ | Clinical data |

## Methods

| n/a | Involved in the study | |
|---|---|---|
| ☒ | ☐ | ChIP-seq |
| ☒ | ☐ | Flow cytometry |
| ☒ | ☐ | MRI-based neuroimaging |
